# Utilization of Crushed Pavement Blocks in Concrete: Assessment of Functional Properties and Environmental Impacts

**DOI:** 10.3390/ma14237361

**Published:** 2021-11-30

**Authors:** Václav Kočí, Jan Kočí, Jan Fořt, Lukáš Fiala, Jiří Šál, Izabela Hager, Robert Černý

**Affiliations:** 1 Department of Materials Engineering and Chemistry, Faculty of Civil Engineering, Czech Technical University in Prague, Thákurova 7/2077, 166 29 Prague 6, Czech Republic; jan.koci@fsv.cvut.cz (J.K.); jan.fort@fsv.cvut.cz (J.F.); fialal@fsv.cvut.cz (L.F.); cernyr@fsv.cvut.cz (R.Č.); 2 Institute of Technology and Business in České Budějovice, Okružní 517/10, 370 01 České Budějovice, Czech Republic; sal@mail.vstecb.cz; 3 Faculty of Civil Engineering, Cracow University of Technology, 24 Warszawska St., 31-155 Cracow, Poland; ihager@pk.edu.pl

**Keywords:** recycled concrete aggregates, material properties, environmental impacts, hygrothermal performance, experimental analysis, computational modelling

## Abstract

Production of concrete is connected to extensive energy demands, greenhouse gases production or primary sources depletion. Reflecting current economical, social, or environmental trends, there is strong pressure on mitigation these requirements and impacts. The exploitation of secondary- or waste materials in production processes has therefore a great potential which is not related solely to binders but also to fillers. In this light, this paper aims at thorough investigations of concrete mixtures with crushed concrete pavements as partial or full replacement of natural coarse aggregates. The research combines experimental techniques to quantify the influence of the substitution on basic physical, mechanical, and heat/moisture transport/storage parameters. The experimental data obtained are further exploited as input data for computational prediction of coupled heat and moisture transport to assess the influence of the aggregates substitution on hygrothermal performance of the built-in concretes. In the last step, the environmental impacts are assessed. Since the changes in the hygrothermal performance were found to be insignificant (i), the compressive strength were improved by up to 25% (ii) and most of the environmental impact indicators were decreased (iii) at the same time, the findings of the research presented predeterminate such a reuse strategy to wider application and use.

## 1. Introduction

Being started by the Portland cement production patented in 1824 [1], concrete has become one of the most popular materials. Since the first batch had been mixed, concrete has made a huge progress that enabled literally unlimited possibilities in practical applications. Impressive mechanical properties, shape variability, availability of raw materials, reasonable price, high performance, or possibility of functional properties tailoring [2,3] can be stated as typical examples in that respect making concrete even more versatile and popular building material all over the world.

Similar to other fields of human activities, concrete industry has to reflect current trends. Being the most topical in the last years, the environmental protection efforts have probably the biggest impact these days. In this context, concrete has been branded as an eco-hostile material whose production goes along with extensive carbon dioxide emissions [4,5], energy consumption [6], and primary sources depletion [7,8]. To reduce the environmental pressure that concrete is exposed to, some measures have been adopted being supposed to make it less harmful to the environment. A partial cement replacement by secondary products such as fly ash, slag, silica fume, and others [8,9] represents the most significant step toward the reduction of energy demands as well as CO_2_ emissions. Utilization of byproducts or even waste materials turned out to be perspective also in case of fillers as it saves primary resources and reduces landfilling necessities, thus generally improving the overall life cycle indicators. Furthermore, from the point of view of the hydration and the structure formation processes, a substitution of the filler is easier as it is not as determining as that of the binder. There are therefore much more possibilities of what can be used [10].

Demolition waste can be considered as a very good candidate that acts like artificial aggregates [11]. According to Jin and Chen [12], up to 7 billion tons of concrete waste is produced every year and this number is still increasing. Being cleaned of impurities, as it might contain fibers or other types of organic and inorganic reinforcement [13], these aggregates exhibit very good mechanical properties, especially when concrete structures are the source of the waste [14]. Concrete slabs, bricks, or blocks from the pavements represent one of the most favorite sources as they are not provided with other constructing layers such as adhesives, renders, or finishes. Moreover, they do not contain any kind of reinforcements. They can be therefore considered as a secondary raw material of high quality, especially when high performance concrete is considered [15].

On the other hand, secondary- and waste materials might go along with some quality issues as their functional properties are usually not stable and may exhibit a significant variance when compared to primary sources. The final products exploiting such materials may then suffer the same issues. Based on that, building materials with waste or recycled components are supposed to represent a compromise between lower functional properties and increased environmental benefits and thus should be assessed in a complex way. A special attention must be also paid to energy demands related to the waste material extraction, purification, and modification prior to its application. Especially the crushing of materials with very high compressive strength (e.g., high performance concrete) might be more energy demanding. On the other hand, it might yield better functional properties.

Such an assessment is the main objective of this paper which aims at developing concrete with aggregates fully or partially replaced with the crushed concrete pavement blocks. The analysis of functional properties together with the quantification of environmental impacts is supposed to answer the question, whether the aforementioned compromise had been reached or one aspect only prevailed the other. Contrary to other paper focusing on that topic, this research is not strictly limited to only functional properties [14,16] or environmental impacts investigations [17], but brings a complex insight to revise the reuse strategy in general. Beside the traditional experimental and environmental impact assessment approaches, the presented paper includes also a computational modelling prediction of the materials performance that should evaluate the usability of the newly developed ecofriendly mixture as a structural material.

## 2. Materials and Methods

### 2.1. Materials

**Figure 1:**
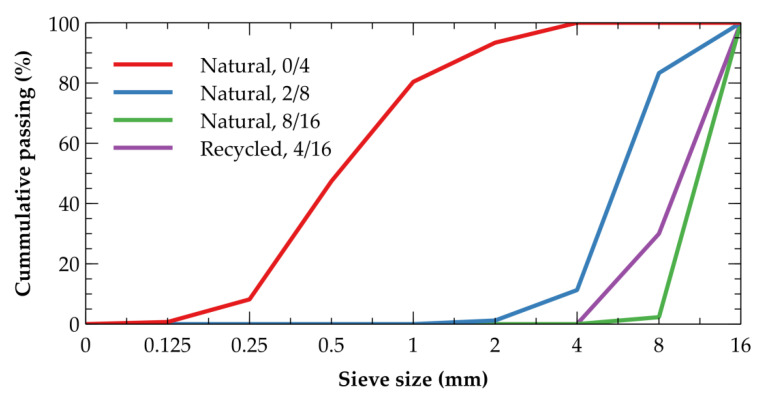
Particle size distribution curves of the aggregates used.

The concrete studied is made of Portland cement CEM I 42.5 R (Malogoszcz, Poland) that is mixed with water to yield the *w*/*c* ratio equal to 0.45. The workability of the mixtures is further increased by an addition of plasticizers BASF BV 18 and BASF Glenium SKY 591. There is also fine and coarse aggregates of which type and amount differ in particular mixtures as summarized in Table 1. The natural aggregates originate from Dwudniaki, Poland (riverbed from Dunajec river), being formed mostly by sandstone (40–50%), granite (15–20%), porfire/melafire (5–30%), and quartzite (5–25%). The recycled aggregates are made of crushed concrete pavement blocks with compressive strength of 66.8 MPa, modulus of elasticity of 41.2 GPa, and bulk density of 2290 kg·m^−3^. The get the better overview, the particle size distribution curves of particular aggregates are depicted in Figure 1. The natural coarse aggregates is replaced either partially (REC50) or fully (REC100), being compared to the reference mixture (REF) without the recycled aggregates being contained.

**Figure 2:**
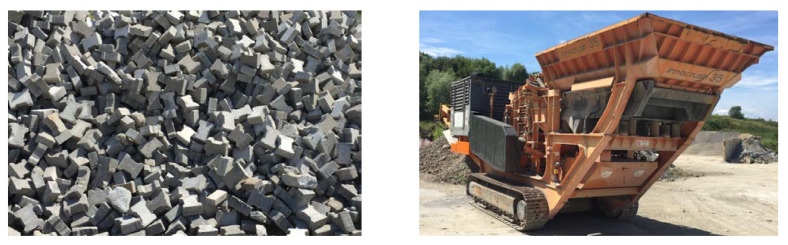
Concrete pavement blocks prior to their recycling (**left**) and the crushing device used for this purpose (**right**).

Recycled aggregate was made simply by crushing the concrete pavement blocks that were dismounted after exploitation period of 15 years (see Figure 2). The only modification was the sieving to achieve the particle size range demanded.

**Figure 3:**
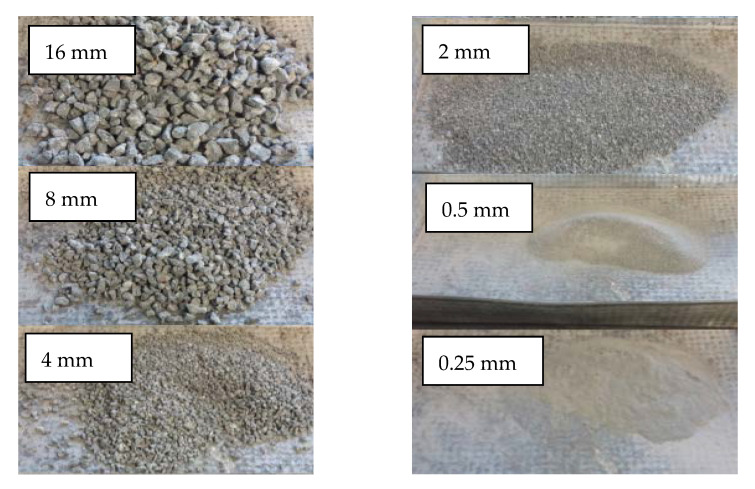
The individual fractions of the recycled coarse aggregates after sieving.

The individual fractions after the crushing and sieving procedures are shown in Figure 3. Accounting for 24.59%, the particles smaller than 4 mm were then rejected.

The concretes specimens were cast and stored in plastic cubic or cylindrical molds for the first 24 h. After preliminary curing, water evaporation was prevented by covering them with plastic lids for 7 days. The specimens were then stored in natural air-drying conditions at the temperature of (20 ± 5) °C and relative humidity of (50 ± 5)%.

### 2.2. Experimental Analysis

Experimental analyses involved a determination of selected basic physical properties as well as heat and moisture storage and transport parameters to evaluate the influence of the recycled aggregates substitution on the functional properties of the concrete.

The bulk density was determined in a standard way [18] by averaging individual values obtained for a set of cubic samples (4 samples of 50 × 50 × 50 mm^3^, 3 samples of 100 × 100 × 100 mm^3^) and a set of prism samples (3 samples of 40 × 40 × 150 mm^3^).

The heat transport and storage parameters represented by thermal conductivity and specific heat capacity, respectively, were obtained by means of transient heat pulse method using the ISOMET 2114 device (Applied Precision, Ltd., Bratislava, Slovakia). Contrary to the steady state methods defined in ČSN EN 12,664 [19], the dynamic method used provides almost instant results which is its biggest advantage. Even if the accuracy is not as high as in case of the standard steady state methods, the probes calibration according to ASTM D5334-14 [20] guarantees the correctness of the data obtained. Being equipped with a surface probe, the sample surface is heated up so that its thermal response can be recorded and evaluated. The measurements were conducted repeatedly on both, dry (nine times) and fully saturated samples (five times).

**Figure 4:**
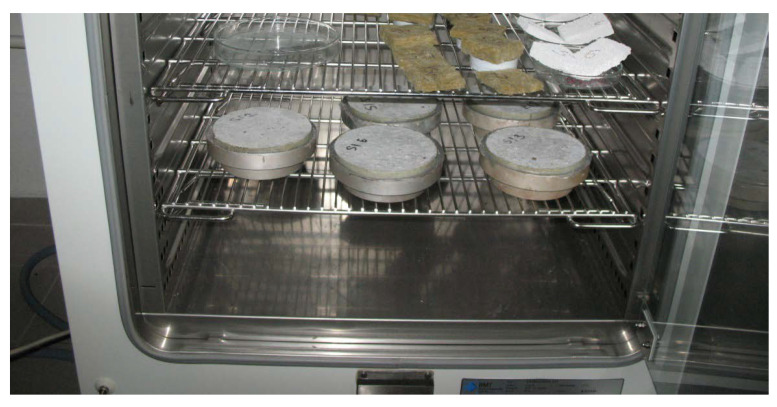
Cup method—placement of cups with samples in a climatic chamber with controlled environment.

Following the principles given in ČSN EN ISO 12,572 [21], the water vapor transport parameters were analyzed and obtained by means of both dry-cup and wet-cup method, as depicted in Figure 4 for illustration.

Three samples of each mixture with dimensions of 100 × 100 × 30 mm^3^ were placed in the cups that contained either water or silica gel, depending on the type of arrangement, and the lateral sides were sealed to enable a one-dimensional moisture flux as the most dominant transport mode. Being placed in the climatic chamber with the controlled environment (25 °C, 50% RH), the water vapor transport had been initiated and the mass change of the cup was continuously recorded. The water vapor diffusion resistance factor could then be expressed as

(1) $$\mu =\frac{D_{a}\cdot M\cdot t\cdot S\cdot \Delta p_{v}}{\Delta m\cdot d\cdot R\cdot T},$$

where *D*_a_ = 2.82 × 10^−5^ m^2^·s^−1^ is the water vapor diffusion coefficient in air at 25 °C, *M* = 0.01802 kg·mol^−1^ is the molar mass of water, *t* (s) is time, *S* (m^2^) is the cross-section area of the sample, Δ*p*_v_ (Pa) is the partial pressure difference above and under the sample, Δ*m* (kg) is the mass change, *d* (m) is the sample thickness, *R* = 8.314 Pa·m^3^·mol^−1^·K^−1^ is the gas constant, and *T* (K) denotes the temperature.

**Figure 5:**
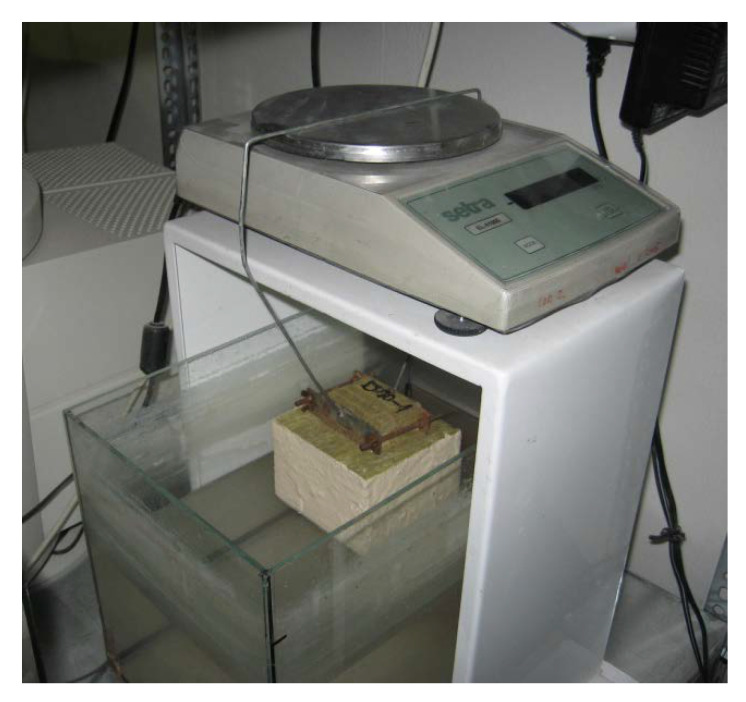
Experimental setup for the automatic measurements of water sorptivity.

Liquid water transport parameters were quantified using moisture diffusivity as a result of the vertical water sorption test [22,23]. Within the experimental procedure, the 50 × 50 × 50 mm^3^ samples insulated on their lateral sides by epoxy resin were partially immersed in water to initiate the water suction due to capillary forces. Based on mass increase observed as a function of time, the apparent moisture diffusivity can be calculated as

(2) $$\kappa_{\mathrm{app}}={\left(\frac{\Delta m}{S\cdot \sqrt{t}\cdot w_{\mathrm{cap}}}\right)}^{2},$$

where Δ*m* (kg) is the mass difference, *S* (m^2^) is the sample area in contact with water, $\sqrt{t}$ (s^0.5^) is time, and *w*_cap_ (kg·m^−3^) stands for the capillary moisture content. The scheme of the experiment is shown in Figure 5.

**Figure 6:**
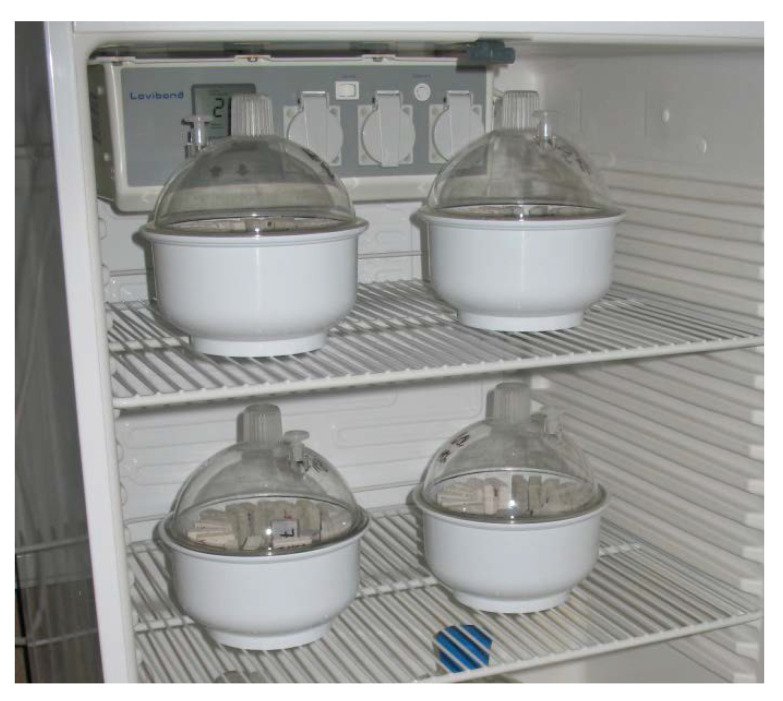
Desiccator method for measurement of the equilibrium moisture content at given relative humidity of environment.

Water vapor storage parameters were represented by the hygroscopic moisture content defining a moisture content threshold after which the moisture is no longer stored in a water vapor- but only in a liquid form. According to common agreement [24] such a threshold corresponds to the equilibrium moisture content at 97% RH. Following this principle, four samples of each mixture were placed in a desiccator with supersaturated solution of K_2_SO_4_ that maintains the humidity of air above the solution at this level (see Figure 6). After the mass equilibrium of the samples had been reached, the moisture content was calculated gravimetrically.

The compressive strength and splitting tensile strength after 28 days and compressive strength after 90 days, were determined following the standard procedures for concrete defined in ČSN EN 12390-3 standard [25].

### 2.3. Hygrothermal Performance

**Figure 7:**
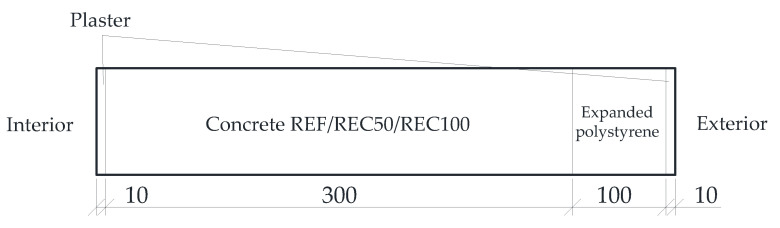
The scheme of the building segment analyzed.

Hygrothermal performance of the mixtures was compared by means of computational prediction of heat and moisture distribution over a reference year after exposure to dynamic weather conditions. A simplified construction segment (wall) was assumed for this purpose, being made of the concrete mixtures studied and provided with thermal insulation and finishes as shown in Figure 7.

The heat and moisture transport was computed using an advanced mathematical model of a diffusion type [26] that is able to precisely distinguish between particular phases of water that participate on the moisture transport at given conditions. The set of balance equation was solved numerically using the finite element method. As the input parameters, the material properties of concrete mixtures obtained experimentally were used, the other parameters of the thermal insulation and the plaster were taken from studies published previously [27,28]. The computational simulation resulted in the determination of hourly values of moisture content and temperature in every node of the computational mesh that together form the temperature and moisture distribution fields and can be further post-processed to get an overview on the hygrothermal performance. The boundary conditions on the exterior side were represented by the test reference year for Ostrava, Czech Republic [29], containing long-term average hourly values of selected weather parameters (temperature, relative humidity, rainfalls, wind velocity, wind direction, direct solar radiation, diffuse solar radiation). The interior boundary conditions were set to 21 °C and 55% of relative humidity according to the thermal standard [30].

### 2.4. Environmental Impact Assessment

The LCA (life cycle assessment) methodology was used to perform the environmental analysis of concrete mixtures studied, which considers each manufacturing step and quantifies all the benefits following the ČSN EN ISO 19,011 [31] and ČSN EN ISO 14,044 [32] standards. All the results presented within this subtask are related to the functional unit which is 1 m^3^.

The analysis considers two scenarios which are combined to accommodate all the mixtures studied [33]. The natural resources scenario contemplates quarrying of granite/limestone, loading, two-stage crushing, loading, and transportation between particular stages. According to Borghi et al. [34], transportation distances for the natural aggregates were assumed to be 30 km. The recycling scenario contemplates the concrete pavement blocks at the end-of-life which must be collected, crushed, and sieved and transported to a concrete plant where it can be used as the filler. Within this scenario, the material needs to be transported about 60 km together including its collection and consequent delivery to the concrete plant [34].

The life cycle impact assessment performed was based on the IMPACT 2002+ methodology, which is very frequently used among researchers and environmentalists (see [35,36]). Following midpoint indicators were used to compare the particular mixtures studied: carcinogens (CA), non-carcinogens (NCA), respiratory organics (RO) and inorganics (RI), aquatic (AE) and terrestrial ecotoxicity (TE), terrestrial acidification/nitrification (TA/N), aquatic acidification (AC) and eutrophication (AEU), land occupation (LO), mineral extraction (ME), non-renewable energy (NRE), ionizing radiation (RI), ozone layer depletion (OLD), and global warming (GW). To depict the environmental burden of materials studied, the endpoint level categories including Human Health, Ecosystem Quality, Climate change, and Resources consumption were determined. All the data gathered to perform the analysis were obtained using the Simapro 8.5 software and Ecoinvent database 3.5.

Since the original material replacement may result in changes in the functional performance, the combined environmental/functional assessment derived from Pedreno-Rojas et al. [37] was employed to access more reliable comparison of studied materials. The overall environmental/functional efficiency, *E*_c_, expresses the environmental costs per unit compressive strength (3), being represented by the weighted endpoint environmental single score:

(3) $$E_{c}=\frac{E}{R_{c}},$$

where *E* (mPt) is the normalized environmental single score, and *R*_c_ (MPa) the compressive strength after 28 and 90 days of curing, respectively.

## 3. Results and Discussion

**Figure 8:**
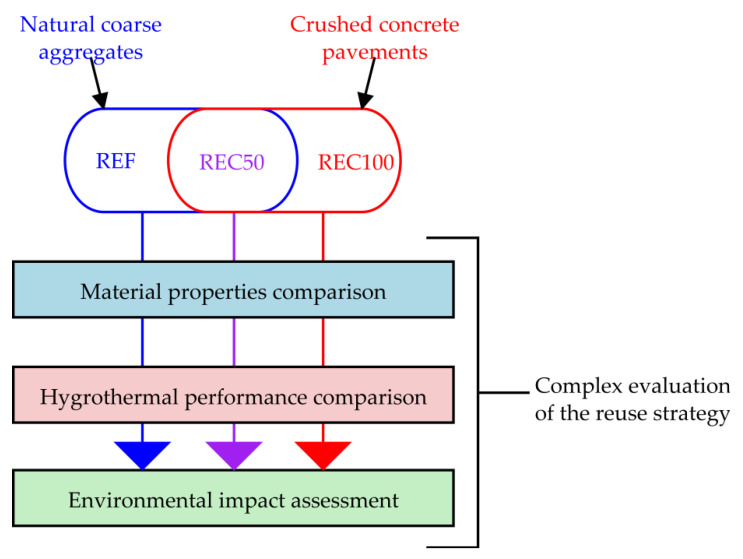
Scheme of the complex assessment of the reuse strategy.

The evaluation of the concrete mixtures with natural coarse aggregates replaced partially or fully is done independently from three different point of views. The individual results are then compared and discussed, forming the complex evaluation of the reuse strategy, as depicted in Figure 8 for a better comprehensibility.

### 3.1. Materials Properties

Only slight changes were found in bulk density values after the coarse aggregate in the reference mixture was replaced. While the bulk density of REF was 2236 kg·m^−3^, the partial- as well as the full replacement of the aggregates resulted in a decrease to 2192 kg·m^−3^, which is less than 2%. The difference might indicate some changes in the structure and the porosity of the REC50 and REC100 samples compared to the REF sample. Being within the measurement uncertainty range (~3%), however, deeper conclusions cannot be formulated based on the difference recognized. Anyway, the very low sorptivity of the natural coarse aggregates from Dwudniaki, Poland (2.5% for 2/8 and 2.3% for 8/16) [38] indicates that this natural raw material is more dense than concrete pavement blocks after 15 years of exploitation (~5% as published by Krawczyk et al. [39]). In this light, the porosity increase and bulk density decrease seems to be logical and justifiable, especially when it correlates to the water absorption values: REF—5.2%, REC50—5.8%, and REC100—6.7%.

The similar trend can be confirmed also by means of a comparison of thermal transport and storage properties, being summarized in Table 2.

Here, the decrease of thermal conductivity of REC50 and REC100, up to 23.7% depending on type of samples and its moisture conditions, can be understood as a clear evidence of the porosity increase. More dense specimens exhibit also higher specific heat capacity, which is also confirmed by the results presented in the table above. Last but not the least, it is important to realize that the differences found are very small which make them prone to be affected by the experimental uncertainties.

The hygric properties of the samples presented in Table 3 might seem contradictory at the first glance when compared to basic physical and thermal properties. Usually, more porous materials exhibit higher permeability or water vapor transport parameters. In this case, however, the water vapor diffusion resistance factor values of REC50 and REC100 samples are higher than those of REC which makes these materials less permeable (see Table 3).

The different water vapor transport properties can be ascribed to various phenomena. It is important to realize that aggregates do not represent primarily the main transport paths for the water vapor. The substitution of the natural aggregates by the crushed concrete should be therefore insignificant, as the bonding matrix covers this type of mass transport. On the other hand, crushed concrete has higher sorption capacity as shown in Table 3, therefore it might partially reduce the amount of water vapor transported through the samples and thus decrease its permeability. Last but not the least, unlike natural aggregates which is inert, the crushed concrete might be still involved in the hydration processes which might result in the formation of more dense structure. A deeper characterization of the bonding matrix as well as an investigation of the pore space characteristics might provide the explanation. Unfortunately, it is beyond the scope of this paper and will be published separately within the future research on this topic. Anyway, it can be stated in general, that all the samples studied are relatively low moisture-conductive as their water vapor diffusion resistance factor is high and the moisture diffusivity values are about ~10^−9^ m^2^·s^−1^.

**Figure 9:**
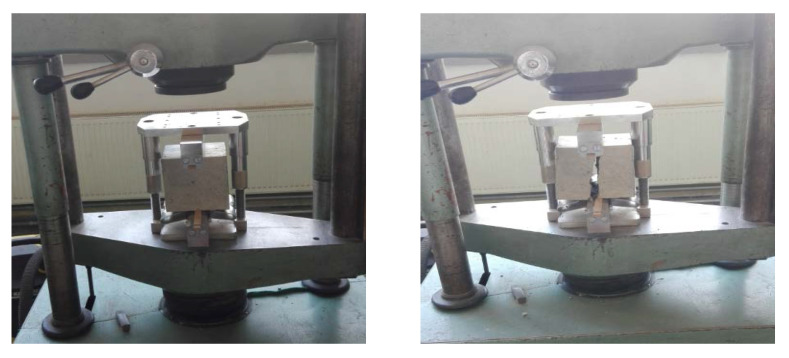
The splitting tensile strength test: a samplebefore **(left)** and after **(right)** the testing procedure.

The procedure of mechanical properties testing, namely the splitting tensile strength test, is depicted in Figure 9 and the results are summarized in Table 4.

**Figure 10:**
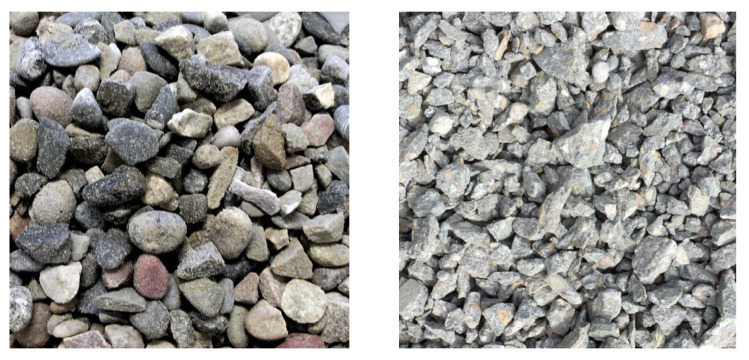
The visual comparison of particles of the natural (**left**) and recycled (**right**) coarse aggregates.

It is clear, that all the mechanical parameters observed were increased after the partial or full replacement of the natural coarse aggregates. The highest increase, ~24.9%, was found in 90-day compressive strength of the REC50. Generally, there are many factors affecting the compressive strength after the natural coarse aggregate substitution and their investigation would require separate research paper as presented e.g., by Xuan et al. [40], which is beyond the scope of this research. In this case, the shape of coarse particles might play a significant role as the crushed recycled coarse aggregates with sharp edges exhibiting lower compressibility contrary to the natural aggregates (riverbed gravel) whose edges are rather round (see Figure 10). The bonding ability of the aggregates to the cement matrix represents another key factor in that respect. It can be expected that the recycled aggregates have a higher bonding potential than the natural aggregates which have significantly smoother surface. Unfortunately, the authors do not possess the SEM images that could support or disprove such a hypothesis.

### 3.2. Hygrothermal Performance

Based on the material parameters summarized in Section 3.1 and their similarity, in particular, it can be expected that the substitution of coarse aggregates is not predisposed to affect the hygrothermal performance of the designed materials significantly. On the other hand, the dynamic boundary conditions on the exterior side of the wall assembly together with different storage and transport properties of particular mixtures can be exhibited unpredictably, so the computational modelling can be used as an effective tool to reveal possible shortages of the materials studied.

**Figure 11:**
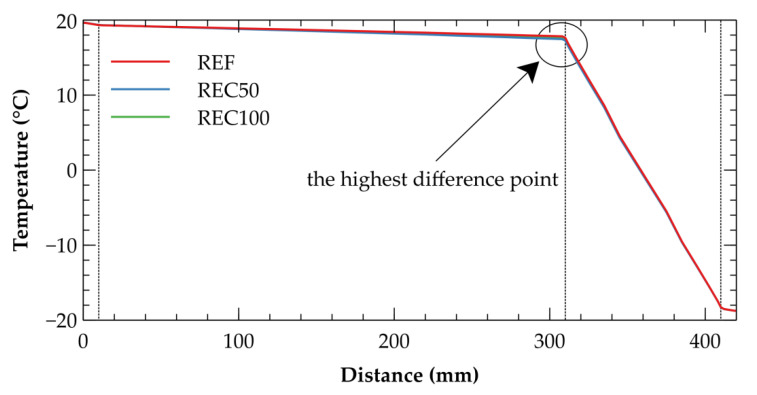
Comparison of temperature profiles as of 12 January of the reference year. The position 0 mm denotes the interior side of the wall, the position 420 mm denotes the exterior side.

Both external layers, the thermal insulation and the exterior finish, assumed in the composition of the wall assembly act like a buffer that mitigates the impacts of weather conditions. In the light of this fact, the thermal performance of the assemblies is almost identical. The highest difference within the reference year was found in a point 110 mm under the surface. As of 12 January of the reference year, this difference was only 0.383 °C as depicted in Figure 11.

**Figure 12:**
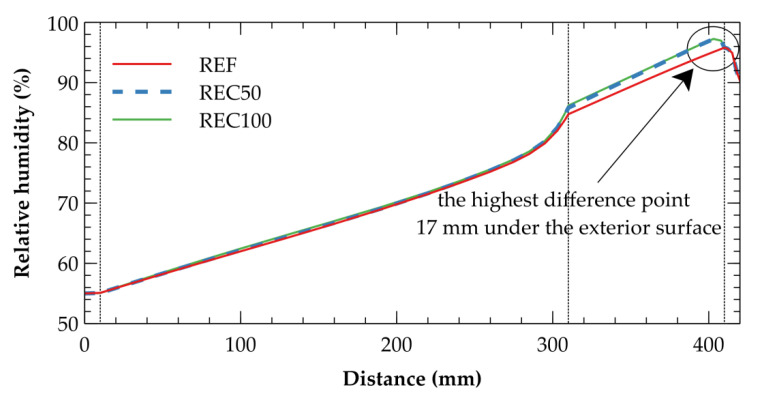
Comparison of relative humidity profiles as of 27 January of the reference year. The position 0 mm denotes the interior side of the wall, the position 420 mm denotes the exterior side.

The hygric performance of the particular assemblies studied differ more than the previously showed temperature performance. However, even in this case, the differences are still very small. The Figure 12 compares the relative humidity profiles as of 27 January of the reference year, where the highest difference was found (2.2%) in the point 17 mm under the exterior surface. Since the outer layers (thermal insulation, plaster) act like a buffer and reduce the response of the base material, the hygrothermal performance of particular concrete mixtures is almost identical and some changes were found only in these layers which are exposed to the dynamic boundary conditions in the most extensive way.

Based on the results of the hygrothermal performance described above it can be concluded that all the mixes studied showed a very similar performance as the highest difference found within the applied reference year was only 0.4 °C and 2.2% of RH, respectively. It means, the substitution of the natural coarse aggregates by the crushed concrete pavements does not have any (negative) effect on the heat and moisture distribution. Such a finding predisposes this measure (aggregates substitution) to be recommended as it might bring other benefits while the performance is not affected. The evaluation of environmental impacts is therefore given in Section 3.3.

### 3.3. Environmental Impact Assessment

Based on the mixtures compositions and the natural-resources/recycling scenarios, the values of particular midpoint indicators are summarized in Table 5.

It can be stated, based on the data presented in Table 3, that utilization of crushed concrete pavements resulted in substantial improvement of most of the indicators evaluated. The highest improvement can be observed in a reduction of aquatic eutrophication and terrestrial acidification/nitrification. A substantial decrease in mineral extraction refers to the preservation of natural resources owing to waste material valorization. Among both recycling scenarios, the complete substitution of the natural aggregates (REC100) provided more favorable results, which can be assigned to less demanding processing. Considering the calculated results for global warming with regard to the carbon dioxide equivalent, all scenarios achieved similar values and only minor improvement was delivered as the process of recycling involves crushing, in particular, energy is very demanding and therefore is negatively reflected in the resulting numbers. On the other hand, only a few indicators refer to an increased negative impact on the natural environment. As concluded by Borghi et al. [34], the replacement of aggregates in concrete does not provide a significant reduction in carbon dioxide production since the longer transportation distance shifted the diesel consumption.

**Figure 13:**
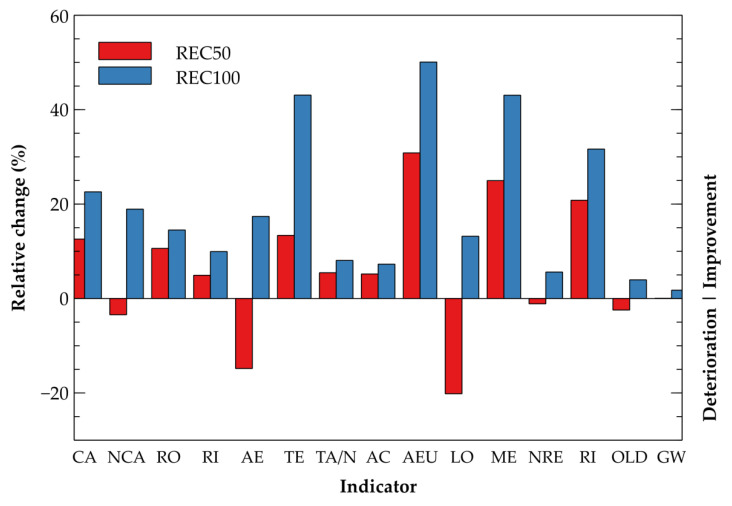
Relative change of the environmental indicators—recycling scenarios.

For a better interpretation, the obtained results are displayed graphically in Figure 13, referring to REF mixture by means of relative change expression.

**Figure 14:**
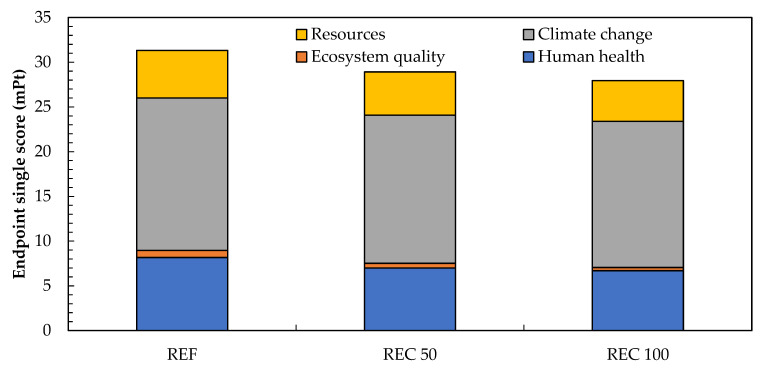
Endpoint categories comparison of the crushed pavements recycling scenarios.

Since the midpoint level consists of many categories, the endpoint comparison is accessed in Figure 14 to depict the environmental implications in a more comprehensible way. As depicted in this figure, the most intense negative consequences of the materials production are associated with climate change. It arises from the energy-demanding production of cement and carbon dioxide emissions during the decomposition of limestone rock. It is obvious, such a share is almost equal for all mixtures. The results of the combined environmental/functional assessment provided in Table 6 show the environmental costs expressed by the cumulative endpoint score per MPa of the compressive strength after 28 and 90 days, respectively. In the light of the improved mechanical performance of designed mixtures REC50 and REC100 with reduced environmental burden, one can see significantly improved environmental efficiency for both mixtures. Here, the environmental costs *E*_c_ was decreased by up to 20% at 28 days and by 17% at 90 days, compared to the reference mixture. Both mixtures REC50 and REC100, differ only very slightly and notable variations were not observed. Contrary to the studies published previously [41,42], the effectiveness of the environmental burden mitigation is more distinct due to concurrent improvements in the mechanical strength and reduced environmental loads.

## 4. Conclusions

The research presented in this paper deals with a complex investigation of concrete mixtures with crushed concrete pavements used as a partial or full replacement of natural coarse aggregates. Such a substitution reflects the main global strategies and efforts that strive to decrease the energy demands, preserve the primary sources, or protect the environment in general to reach sustainable development. Within the light of the aforementioned efforts, this research aims at a thorough investigation of the mixtures designed to assess their material properties and hygrothermal performance as well as to quantify their contribution to mitigation of negative environmental impacts.

The thorough experimental analyses of basic physical, heat and moisture transport, and storage parameters revealed that the substitution of the natural coarse aggregates for crushed concrete pavements at the end of their service life did not influence the material properties in a negative way. It was found, on the contrary, that an increase of porosity was positively reflected in a decrease of thermal conductivity of the new mixtures by up to 23.7%, which predisposed them to achieve better thermal performance when used e.g., in building constructions. On the other hand, such an advantage can be neglected by the presence of thermal insulation in the assembly as demonstrated in the example exploiting computational modelling of coupled heat and moisture transport to predict the hygrothermal performance of construction segments. Here, the highest differences between particular materials accounted only for 0.4 °C and 2.2% of relative humidity, respectively, within a reference year. It indicates, in any case, that the hygrothermal performance of the concrete is not negatively affected after the aggregates substitution.

In the last field of the investigation, the analysis of environmental impacts confirmed a positive contribution of the recycling scenarios to the environmental protection as most of the indicators observed showed favorable changes. The most significant contribution can be found, among others, in mitigation of aquatic eutrophication (by up to 50.08%), terrestrial ecotoxicity (by up to 43.08%), or mineral extraction (by up to 43.06%). In general, the full replacement scenario showed more valuable results than the partial replacement.

Based on the complex assessment (material properties, hygrothermal performance, and environmental analysis) of concrete with various levels of crushed concrete pavements it can be concluded that the full replacement of natural coarse aggregates does not negatively affect neither their material properties nor their hygrothermal performance. On the other hand, since this measure goes along with positive changes in environmental impacts, crushed concrete pavements as the filler can be highly recommended. The results obtained proved that utilization of crushed pavements as natural aggregate replacement provides notable benefits in terms of sustainable assessment of building materials as described before. Notwithstanding, our study points to the overall favorable effects associated with crushed pavements reuse. Improvements in the mechanical strength together with lowered environmental impact predeterminate such reuse strategy to wider application and use.

## Figures and Tables

**Table 1:** Summary of mixture compositions.

	REF	REC50	REC100
CEM I 42.5 R (kg·m^−3^)	414.2	414.2	414.2
Water (kg·m^−3^)	186.4	186.4	186.4
Plasticizer BASF BV 18 (wt.%)	0.9	0.9	0.9
Plasticizer BASF Glenium Sky 591 (wt.%)	1.4	1.4	1.4
Natural fine aggregates 0/4 (kg·m^−3^)	615.1	280.6	569.2
Natural coarse aggregates 2/8 (kg·m^−3^)	579.9	280.6	---
Natural coarse aggregates 8/16 (kg·m^−3^)	562.3	264.1	---
Recycled concrete aggregates 4/16 (kg·m^−3^)	---	825.3	1057.2

**Table 2:** Thermal transport and storage properties.

		REF	REC50	REC100
Thermal conductivity (W·m^−1^·K^−1^)	dry	2.41	1.84	2.08
	saturated	2.79	2.30	2.29
Specific heat capacity	dry	817	785	812

**Table 3:** Moisture transport and storage properties.

		REF	REC50	REC100
Water vapor diffusion resistance factor (-)	dry-cup	89.3	124.3	131.0
	wet-cup	42.9	58.6	66.6
Sorption capacity at 97% (m^3^·m^−3^)		9.09	10.99	10.49
Moisture diffusivity (m^2^·s^−1^)		7.13 ×·10^−9^	1.58 ×·10^−8^	1.46 ×·10^−9^

**Table 4:** Mechanical properties.

		REF	REC50	REC100
Compressive strength (MPa)	28 days	46.1	56.8	54.5
	90 days	52.2	65.2	64.0
Splitting tensile strength (MPa)		3.3	3.6	3.4

**Table 5:** Results of assessment of environmental impacts—selected midpoint indicators.

	Unit	REF	REC50	REC100
Carcinogens (CA)	kg·C_2_H_3_Cl·eq	0.207928	0.181762	0.160954
Non-carcinogens (NCA)	kg·C_2_H_3_Cl·eq	0.376624	0.389560	0.305319
Respiratory organics (RO)	kg·C_2_H_4_·eq	0.080252	0.071736	0.06861
Respiratory inorganics (RI)	kg·PM_2.5_·eq	0.032686	0.031078	0.029436
Aquatic ecotoxicity (AE)	kg·TEG·water	1235.460	1418.594	1020.719
Terrestrial ecotoxicity (TE)	kg·TEG·soil	915.9246	793.4930	521.3207
Terrestrial acidification/nitrification (TA/N)	kg·SO_2_·eq	2.372920	2.243352	2.181528
Aquatic acidification (AC)	kg·SO_2_·eq	0.489180	0.463767	0.453614
Aquatic eutrophication (AEU)	kg·PO_4_·P-lim	0.002552	0.001765	0.001274
Land occupation (LO)	m^2^·a	0.936772	1.125691	0.813272
Mineral extraction (ME)	MJ·surplus	0.686285	0.514924	0.390787
Non-renewable energy (NRE)	MJ·primary	807.8142	816.8235	762.527
Ionizing radiation (RI)	Bq·C^−14^·eq	384.3772	304.4095	262.7194
Ozone layer depletion (OLD)	kg·CFC^−11^·eq	9.86 × 10^−6^	1.01 × 10^−5^	9.47 × 10^−6^
Global warming (GW)	kg·CO_2_·eq	168.6626	168.5723	165.6829

**Table 6:** Results of the combined environmental/functional performance.

Mixture	Environmental Costs *E*_c_ (mPt/MPa)	
	28 Days	90 Days
REF	0.6793	0.5999
REC50	0.5091	0.4435
REC100	0.5128	0.4366

## Data Availability

The data presented in this study are available on request from the corresponding author.
